# The Intra- and Inter-Rater Reliability of a Variety of Testing Methods to Measure Shoulder Range of Motion, Hand-behind-Back and External Rotation Strength in Healthy Participants

**DOI:** 10.3390/ijerph192114442

**Published:** 2022-11-04

**Authors:** Peter Beshara, Ingrid Davidson, Matthew Pelletier, William R. Walsh

**Affiliations:** 1 Department of Physiotherapy, Prince of Wales Hospital, Sydney, NSW 2031, Australia; 2 Prince of Wales Clinical School, Faculty of Medicine, University of New South Wales, Sydney, NSW 2031, Australia; 3 Surgical & Orthopaedic Research Laboratories, Prince of Wales Hospital, Sydney, NSW 2031, Australia

**Keywords:** shoulder, range of motion, strength, dynamometer

## Abstract

This study determined the intra- and inter-rater reliability of various shoulder testing methods to measure flexion range of motion (ROM), hand-behind-back (HBB), and external rotation (ER) strength. Twenty-four healthy adults (mean age of 31.2 and standard deviation (SD) of 10.9 years) without shoulder or neck pathology were assessed by two examiners using standardised testing protocols to measure shoulder flexion with still photography, HBB with tape measure, and isometric ER strength in two abduction positions with a hand-held dynamometer (HHD) and novel stabilisation device. Intraclass correlation coefficient (ICC) established relative reliability. Standard error of measurement (SEM) and minimum detectable change (MDC) established absolute reliability. Differences between raters were visualised with Bland–Altman plots. A paired t-test assessed for differences between dominant and non-dominant sides. Still photography demonstrated good intra- and inter-rater reliability (ICCs 0.75–0.86). HBB with tape measure demonstrated excellent inter- and intra-rater reliability (ICCs 0.94–0.98). Isometric ER strength with HHD and a stabilisation device demonstrated excellent intra-rater and inter-rater reliability in 30° and 45° abduction (ICCs 0.96–0.98). HBB and isometric ER at 45° abduction differed significantly between dominant and non-dominant sides. Standardised shoulder ROM and strength tests provide good to excellent reliability. HBB with tape measure and isometric strength testing with HHD stabilisation are clinically acceptable.

## 1. Introduction

Reliable measurements of shoulder joint range of motion (ROM) and strength are essential during a clinical examination to diagnose shoulder pathology, evaluate treatment efficacy and quantify changes in joint mobility or muscle force [1,2,3]. Clinicians must be convinced that a change in ROM or strength is due to a genuine change in the patient’s status rather than an error in measuring instruments or methods, especially in patients with chronic health conditions who have repeated measures taken over time [4].

A variety of methods for measuring shoulder joint ROM include goniometry [5,6], visual estimation [7,8], inclinometry [9,10], tape measure [11,12], still photography [13,14], inertial sensors [15,16], smartphone devices [17,18] and markerless 3D motion-tracking systems [19,20]. Goniometry is widely used in clinical settings to quantify ROM since it is affordable and portable. However, using two hands in goniometry makes it difficult to stabilise the joint properly and increases the risk of measurement error [21]. Furthermore, the time taken to accurately position the goniometer against the anatomical landmarks of the shoulder can further exacerbate the patient’s symptoms.

Still photography offers the benefit of reducing measurement errors and patient discomfort when evaluating shoulder ROM. Several studies have shown that photography-based goniometry is clinically valid for measuring elbow, shoulder abduction, and internal rotation ROM [22,23,24,25]. In addition, photography has shown excellent intra-rater reliability for shoulder ROM measurements, with intra-class correlation values (ICCs) ranging from 0.88 to 0.98 [12,13].

Tape measurement for hand-behind-back (HBB; shoulder internal rotation/adduction with elbow flexion) has been validated with higher accuracy than the vertebral-level method (i.e., identifying the highest vertebral level the thumb can reach by palpating bony landmarks) [11]. However, tape-based methods exhibit wide variations in intra-rater reliability (ICCs 0.016–0.91) and inter-rater reliability (ICCs 0.12–0.80) [26,27,28]. HBB is a challenging movement to measure because it can be difficult to identify and palpate spinal landmarks. van den Dolder and Roberts [28] devised a modified tape measure technique for HBB measurement that demonstrated high reliability (ICCs 0.95, 0.96) by using the posterior superior iliac spines (PSIS) as body landmarks [29]. In contrast to van den Dolder et al. [29], we used two examiners with differing years of clinical experience (one senior, one junior) to assess the protocol’s reliability.

Hand-held dynamometry (HHD) is considered a valid tool for assessing muscle strength that bridges the gap between subjective manual muscle testing and “gold standard” isokinetic testing [2,30,31,32]. According to several studies, HHD has excellent intra- and inter-rater reliability for isometric strength assessment of the shoulder joint [33,34,35,36]. However, tester strength [37,38], inadequate standardisation methods [39,40], and a lack of stabilisation [41,42] can significantly impact the reliability of the HHD.

Kolber et al. [43] utilised a PVC-made stabilising device to measure isometric shoulder external and internal rotation to overcome the limitations of HHD and improve accuracy. The protocol in the study required the examiner to kneel on the floor and manually hold the pipe against the wall throughout the assessment. We designed a hands-free, portable, height-adjustable, novel stabilisation device to alleviate the therapist’s measurement burden, enhance stability and provide a clinically feasible alternative for using HHD to measure isometric external rotation (ER). We hypothesised that our stabilisation device compared to the one in Kolber et al. would demonstrate similar or higher ICC values for reliability. Additionally, we used the Kolber et al. protocol to determine inter-rater reliability and assess isometric ER strength in 45° abduction, both of which had never been studied previously.

A review by Makhni et al. [44] found significant variability in reporting ROM and strength outcomes after rotator cuff tear and repair, including in high-impact orthopaedic journals. Therefore, an assessment of reliability is vital to ensure the same instrument delivers a stable and consistent score when used repeatedly. Accurate shoulder measurements that use reliable and valid tools enable better interpretation of research findings and comparisons of outcomes between studies.

The aim of this study was to examine and verify the intra- and inter-reliability of previously published testing protocols to measure shoulder isometric ER strength, forward flexion ROM and HBB, using a variety of different tools and shoulder positions.

## 2. Materials and Methods

### 2.1. Participants

A convenience sample of 24 asymptomatic adults (12 male and 12 female) with pain-free upper extremity function and no known history of shoulder or neck pathology were recruited from a public hospital in Sydney, Australia (Prince of Wales Hospital Physiotherapy department). Study participant characteristics are reported in Table 1. Before assessment, all participants read and signed a written consent form. This study was approved by South Eastern Sydney Local Health District Ethics Committee (ref no. 14/040).

A priori sample size calculation was based on the methods of Walter et al. [45], assuming a significance level (α) = 0.05, probability of type II error (β) = 0.2, minimal acceptable level of reliability (ρ0) = 0.7, and expected reliability (ρ1) = 0.9, a sample size of 18 participants would be required.

### 2.2. Raters

Rater A and Rater B were registered physiotherapists at Prince of Wales Hospital with 10 years and 2 years of rotating hospital clinical experience, respectively. Both raters were blinded to the muscle strength data, and a third independent unblinded rater recorded all measurements. Rater A was male, height 1.69 m; and weight, 72 kg; and Rater B was female, height, 1.68 m; weight, 56 kg.

### 2.3. Instruments

Shoulder flexion ROM was measured using a tablet device (Samsung Tab S, Samsung, Seoul, South Korea) with an 8-megapixel camera and 8.4” Super AMOLED touchscreen with a 2560 × 1600 pixel resolution. The degrees were obtained using a small (15 cm), two-armed plastic transparent Baseline goniometer (Gymna International, Bilzen, Belgium), with a 360° head and angle scale of 1° increment. HBB measurements were measured using a tape measure.

**Figure 1:**
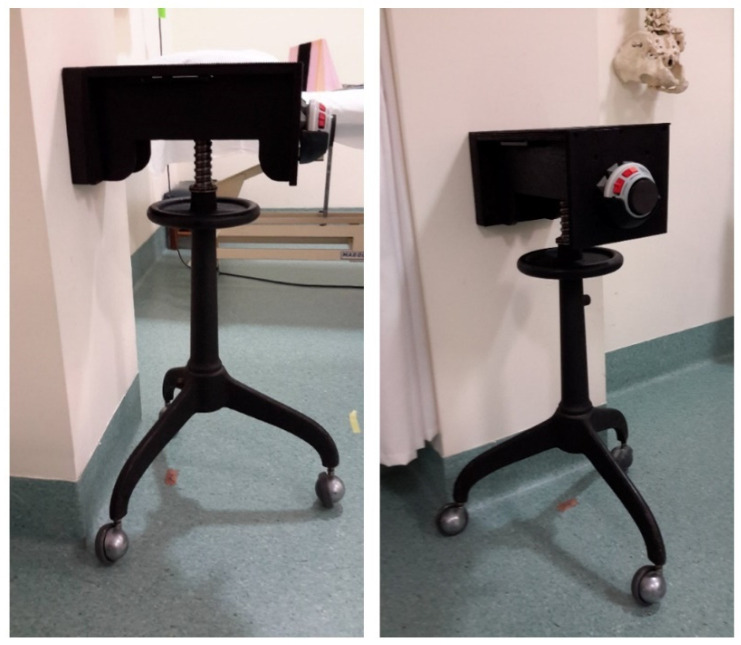
The height-adjustable stabilisation device with HHD inserted and the 3-wheel casters fixed to the floor.

Isometric muscle strength of the shoulder external rotators was measured using the MicroFET 2 HHD (Hoggan Scientific, Salt Lake City, UT, USA). The MicoFET2 HHD is a portable, battery-operated load cell system that records strength between 0.8 and 100 pounds. A digital screen displayed the peak force at low or high thresholds, with a low threshold selected for better sensitivity. A hospital engineer designed a novel stabilisation device (Figure 1) to enhance HHD measurement accuracy. A slot on the side of the device was built to fit the HHD dimensions, and a rotatory wheel allowed correct height modifications depending on the patient. Like Kolber et al. [43], investigators used a plastic hinged-arm apparatus to maintain 30° and 45° of abduction in the scapular plane.

### 2.4. Procedures

Raters A and B measurements were taken independently on separate days, with repeat testing occurring after 14 days. The raters had no contact during the assessments, and any residual markings on the patient’s skin were removed at the end of the testing. All measurements were taken bilaterally, and identical procedures were used for sessions 1 and 2. The measurement results were recorded on paper and stored on an excel spreadsheet by a third independent rater.

**Figure 2:**
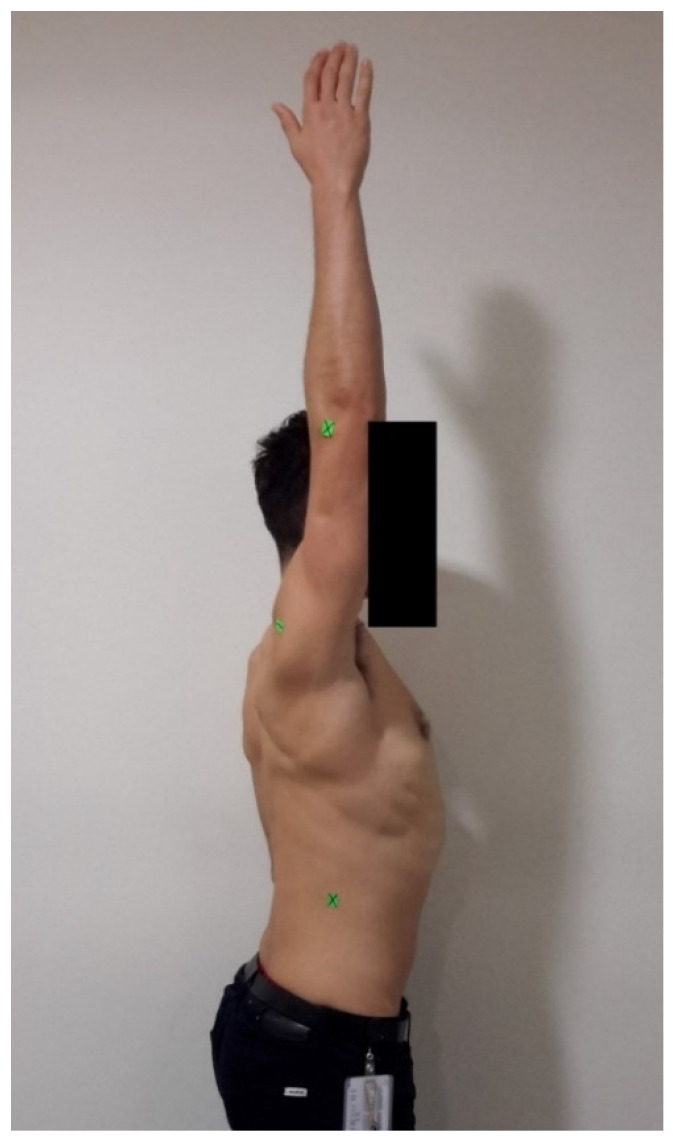
Landmarks for forward flexion ROM as per Ginn et al. [12].

All participants completed a simple, standardised warm-up procedure of general shoulder movements prior to assessment. The procedure started with shoulder forward flexion, using the identical protocol as Ginn et al. [12]. Reference points were identified using the following landmarks: the lateral epicondyle of the humerus, the point 6cm below the postero-lateral acromion process and the end of rib 12 (Figure 2). To ensure consistency and control for potential errors due to positioning, participants stood at a position marked by tape on the floor that was 1.5 metres away from the camera. The backdrop was a plain white wall with no decorations. The participant was instructed to move the upper extremity (thumb pointing upwards) to the end of active shoulder flexion within comfort levels. The assessor used the tablet device to photograph each end-of-range position from a perspective aligned with the axis joint of motion. The digital photograph was taken of the patient in profile, with the camera lens positioned parallel to the sagittal plane at eye level. All images were saved in JPEG format (1836 × 3264 pixels). A standardised, full-print A4 size (21 cm × 29.7 cm) colour photo was printed for each participant. Using a plastic goniometer and the printed photograph, the assessor manually calculated the resultant joint angle in degrees.

**Figure 3:**
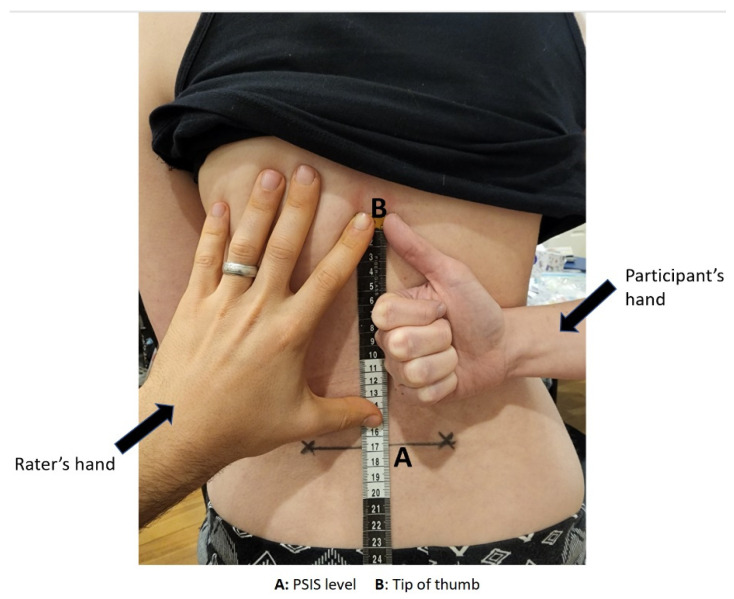
HBB testing position adapted with permission from van den Dolder et al. [29]. 2014, Churchill Livingstone.

HBB was measured using a tape measure and non-permanent marker with the identical testing protocol of van den Dolder et al. [29]. The raters identified each PSIS on the participant and connected both by drawing a horizontal line. Participants were instructed to “take your arm as far as you can go,” and the distance between the level of the PSIS and thumb tip was measured in centimetres (Figure 3). The difference in ROM between the dominant and non-dominant sides was recorded, with a lower number indicating a poorer ROM. If a participant could not reach the PSIS level, the distance from that point was recorded as a negative value.

Isometric ER muscle strength was assessed with a HHD and stabilisation device using the same testing protocol as Kolber et al. [43]. The dynamometer and stabilisation device were set against a wall at an appropriate height for each participant. This height was recorded and standardised for all follow-up assessments.

**Figure 4:**
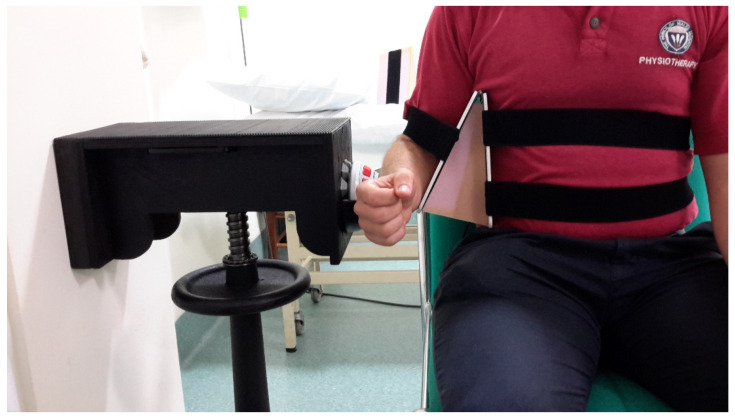
Isometric ER strength testing position with the arm apparatus under the patient’s axilla and an inserted foam wedge for 30° abduction as per Kolber et al. [43].

Participants were seated in an armless chair with their trunk supported and feet flat on the floor. A hinged arm apparatus was positioned beneath the participant’s axillary area, and separate foam wedges were inserted to keep the humerus at 30° and 45° in the scapular plane. Velcro straps attached to the arm apparatus were fastened around the trunk and humerus to prevent unwanted active abduction during testing (Figure 4).

To confirm the correct technique was performed, participants made one isometric contraction (i.e., push against resistance) against the assessor’s hand. Participants positioned the arm in 0° rotation, and 90° elbow flexion with the wrist in neutral. In each trial, participants pushed on the dynamometer’s circular padded contact surface using the distal aspect of their radius/ulnar. Participants were instructed to apply their “maximum and best” effort to the device for six seconds.

Raters gave no verbal encouragement, and all instructions were standardised. Strength was measured three times, with a fourth measurement taken if the third trial exceeded the second. A 10-s rest interval was provided between trials, and the maximum force output from the trials was used in the analysis.

### 2.5. Statistical Analysis

Descriptive data, including means and standard deviations (SD) were calculated for all measurements, with ROM values in degrees and centimetres and isometric strength data in kilograms.

Reliability was determined using ICCs with corresponding 95% confidence intervals (CI) [46]. For intra-rater reliability, an ICC (2.1) two-way random model for absolute agreement was used because a single rater was the only rater of interest. Inter-rater reliability used an ICC (2.2), two-way mixed model, to estimate the absolute agreement between two independent assessors [47].

Reliability was assessed using the criteria by Portney and Watkins [47], where ICC values above 0.90 were considered excellent reliability, 0.75 to 0.90 were good, and less than 0.75 were moderate to poor.

Since ICCs can be impacted by intersubject variability, absolute measures of reliability were determined [48]. The standard error of measurement (SEM) is the amount considered as measurement error, with a lower SEM indicating high reliability. The SEM was calculated with the equation: SEM = SD × $\sqrt{1-\mathrm{ICC}}$ [47]. By determining the SEM, the minimal detectable change (MDC) was calculated with the equation: MDC = SEM × 1.65 × √2. The MDC is the smallest change needed to be confident that a change between two tests is a “true” change and not due to measurement error [47]. Bland–Altman plots [49] were utilised to visualise inter-rater reliability with 95% limits of agreement (LoA) calculated with the equation: 95% LoA = mean difference ± 2SD.

A paired *t*-test was used to calculate the differences between dominant and non-dominant side for Raters A and B. Peak measurement comparisons between participants’ dominant and non-dominant arms for shoulder flexion ROM, HBB and ER strength were also reported for both raters.

## 3. Results

### 3.1. Intra-Rater Reliability

Results of intra-rater reliability with ICC, SEM and MDC values are presented in Table 2. For each rater, still photography demonstrated good intra-rater reliability (ICCs 0.76, 0.86) for ROM assessment of active shoulder forward flexion and excellent intra-rater reliability (ICCs 0.94, 0.96) for HBB tape measurements.

Isometric ER strength measurements with HHD and the stabilisation device demonstrated excellent intra-rater reliability in 30° abduction (ICCs 0.97, 0.96) and 45° abduction (ICCs 0.97, 0.98). SEM and MDC95 values were relatively low across all measurement methods, indicating good absolute reliability. Table 3 shows the mean and SDs for both raters over both sessions.

### 3.2. Inter-Rater Reliability

Results of inter-rater reliability with ICC, SEM and MDC_95_ values are presented in Table 4. Inter-rater reliability for shoulder flexion assessment with photography was good (ICC = 0.75) and excellent (ICC = 0.91) for HBB with tape measure. Similarly, inter-rater reliability was excellent for isometric ER strength measures in 30° abduction (ICC = 0.97) and 45° abduction (ICC = 0.98).

**Figure 5:**
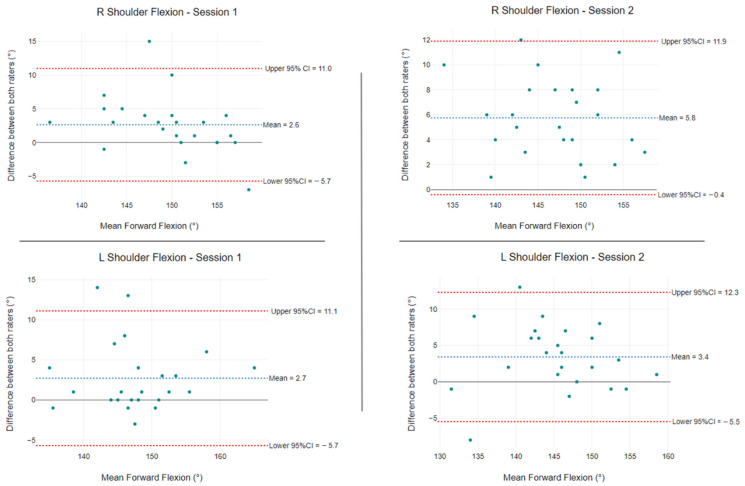
Shoulder flexion ROM differences between raters for left and right sides (n = 24). The blue segmented line is the mean difference between raters, and the segmented red lines show the 95% LoA.

**Figure 6:**
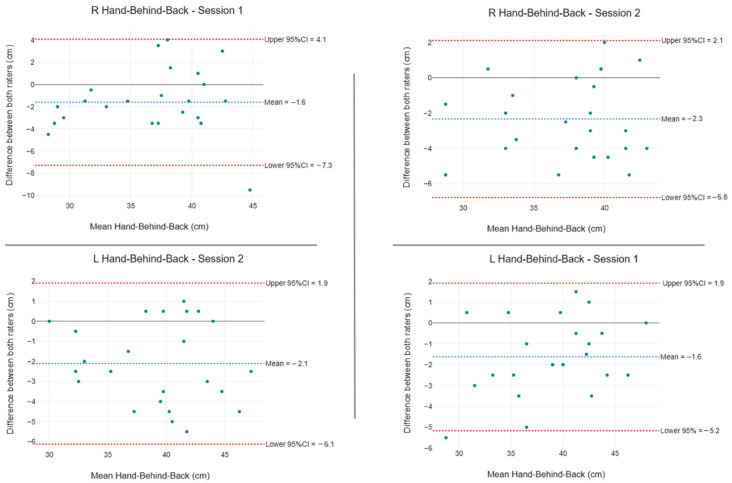
HBB differences between raters using tape measure for left and right sides. The blue segmented line is the mean difference between raters, and the segmented red lines show the 95% LoA.

**Figure 7:**
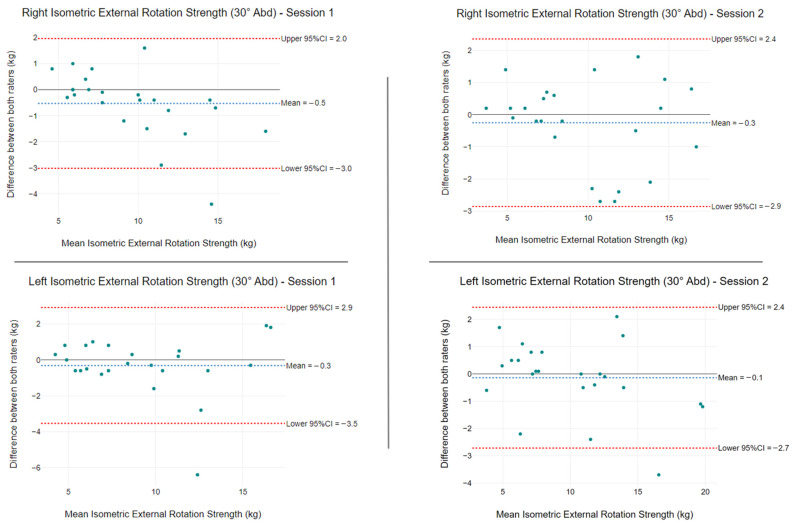
Isometric shoulder ER strength at 30° of abduction differences between raters using a HHD and stabilisation device) for left and right. The blue segmented line is the mean difference between raters, and the segmented red lines show the 95% limits of agreement.

**Figure 8:**
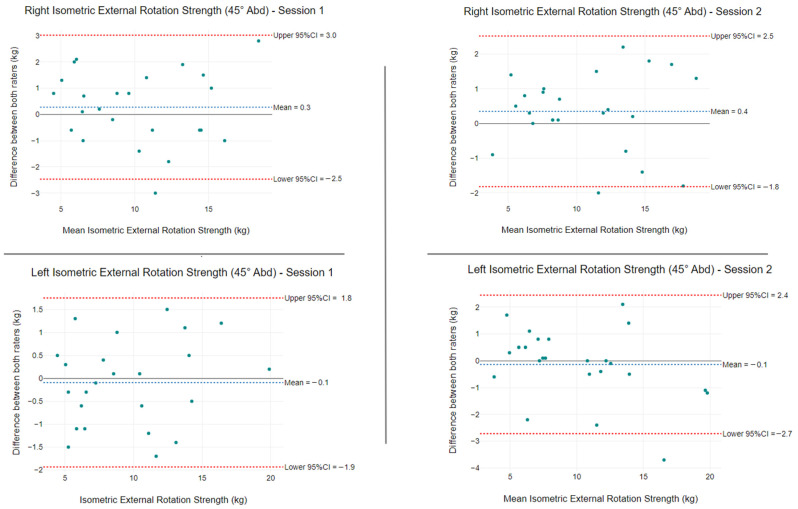
Isometric shoulder ER strength at 45° of abduction differences between raters using a HHD and stabilisation device) for left and right. The blue segmented line is the mean difference between raters, and the segmented red lines show the 95% limits of agreement.

Bland–Altman plots for ROM (Figure 5 and Figure 6) and strength (Figure 7 and Figure 8) were used to evaluate the level of agreement and bias between the mean differences for both raters. The mean shoulder forward flexion differences between raters were 2.6° and 2.7° for session one and 5.8° and 3.4° for session two (Figure 5). The 95% CI LoA ranged from −5.7° to 11.0° and −5.7° to 11.1° for session one, and −0.4° to 11.9° and −5.5° to 12.3° for session two (Figure 5).

The mean HBB differences between raters were −1.6 cm and −2.1 cm in session one and −2.3 cm and −1.6 cm in session two. All ROM values were distributed sparsely and not strongly concentrated along the horizontal axis (Figure 6).

At 30° of abduction, the mean isometric ER strength differences between raters were −0.5 kg (LoA: −3.0 kg to 2.0 kg) and −0.3 kg (LoA: −3.5 kg to 2.9 kg) for session one, and -0.3 kg (LoA: −2.9 kg to 2.4 kg) and −0.1 kg (LoA: −2.7 kg to 2.4 kg) for session two (Figure 7).

At 45° of abduction, the mean isometric ER strength differences between raters were 0.3 kg (LoA: −2.5 kg to 3.0 kg) and −0.1 kg (LoA: −1.9 kg to 1.8 kg) for session one, and 0.4 kg (LoA: −1.8 kg to 2.5 kg) and −0.1 kg (LoA: −2.7 kg to 2.4 kg) for session two (Figure 8).

### 3.3. Comparisons between the Nominant and Non-Dominant Arm

Table 5 compares the shoulder measurements of Raters A and B based on hand dominance. There were significant differences between dominant and non-dominant sides for HBB (−2.2 cm, 2.7 cm, *p* = < 0.001) and isometric ER strength at 45° abduction (0.7 kg, *p* = < 0.001; 0.5 kg, *p* = < 0.011) for both rater assessments. For shoulder flexion ROM assessments, no statistically significant differences in arm dominance were identified.

The peak measurement comparisons for the dominant and non-dominant arms are presented in Table 6. For each session, both raters observed that the dominant arm produced the highest peak forward flexion, HBB and isometric ER strength at 30° and 45° abduction. Non-dominant arm peak measurements were higher for forward flexion ROM and isometric ER strength than HBB. For both raters in any session, only a limited number (<10%) of participants generated peak measurements equally for both arms for any movement. When comparing isometric testing positions, raters observed that the dominant arm was predominately strongest for ER at 30° and 45° abduction.

## 4. Discussion

In this study, we used established testing protocols to determine good reliability for photographic evaluation of shoulder flexion ROM and excellent reliability for HBB assessment with a tape measure. Similarly, HHD with a stabilising device produced excellent intra- and inter-rater reliability when evaluating isometric strength, irrespective of abduction position.

When compared to other types of measurement, the still photography method had the lowest ICC value (<0.90) for both raters when quantifying shoulder flexion. Prior studies using identical testing protocols for still photography reported ICC values of 0.88 [12] and 0.92 to 0.98 [13]. In our study, still photography with a tablet device resulted in good ICC values of 0.86 and 0.75 with wide 95% CIs. In agreement with our findings, Hayes et al. [21] employed the same photographic testing protocol and reported an ICC of 0.75 for shoulder flexion ROM. The Bland–Altman plots for still photography demonstrated low mean differences but wide 95% LoA above the clinically significant threshold of 5° [50].

HBB measurement using the modified PSIS technique with tape measure had excellent intra-rater reliability (ICCs 0.96, 0.94) and inter-rater reliability (ICC = 0.91). Our findings support those of van den Dolder et al. [29], who reported intra-rater reliability ICC values of 0.95 and inter-rater reliability ICC values of 0.96. Similarly, we found small SEM and MDC values. For the HBB protocol, we found that raters with 2 or 10 years of clinical experience were just as reliable as examiners with 15 years of musculoskeletal experience, as used by van den Dolder et al. [29]. Bland–Altman plots found no uniformity or systematic measurement differences.

Isometric ER strength measurements demonstrated excellent intra- and inter-rater reliability for the two abduction positions tested. Using a hinged arm apparatus that allows testing in varying degrees of abduction with a foam wedge insertion, we modified the prior method by Kolber et al. [43]. The original testing protocol placed the patient at 30° abduction, with the authors citing reduced capsular stress, appropriate muscle length tension, and preventing hypovascular adducted positions as reasons [51,52,53]. In accordance with findings by Edouard et al. [54], we also tested ER in the seated position at 45° abduction in the scapular plane, as this position was reported to be most reliable. Our study established that both positions produced excellent intra- and inter-rater reliability, with ICC values ranging from 0.96 to 0.98. Only Rater B produced a higher reliability for ER strength at 45° of abduction (ICC = 0.98). A systematic review by Schrama et al. [55] evaluated intra-rater reliability for manual HHD and reported ICCs ranging from 0.77 to 0.98 for ER strength measures. In contrast to our study, Hayes et al. [32] observed excellent intra-rater reliability (ICC 0.92) and good inter-rater reliability (ICC 0.82) with manual HHD to quantify isometric ER strength in symptomatic patients.

Although it was not the focus of our study, both raters identified statistically significant differences in dominant and non-dominant arms for HBB and isometric ER strength at 30° abduction. Peak measurement values were greater in the dominant arm for all measurements for both raters. The non-dominant arm was most common for peak forward flexion.

Despite evidence that still photography produces good reliability and less potential for exacerbating shoulder symptoms, the technique is more time-consuming than standard goniometry. Marking bony landmarks, precisely positioning the patient for the photograph, and printing the photo to manually calculate the angle all take a considerable amount of time. While still photography is useful for research purposes, it is impractical in a clinical setting.

Conventional goniometry to measure active shoulder flexion ROM in asymptomatic shoulders demonstrates acceptable intra-rater reliability (ICCs 0.86–0.97) [56,57,58] and inter-rater reliability (ICCs 0.69–0.79) [21,56,58]. Wearable inertial sensor technologies, smartphone-based applications, and optical motion capture systems have recently emerged as more accurate, valid, and reliable methods of assessing shoulder ROM [59,60,61].

HHD with a stabilisation device demonstrates high reliability for ER and is a cheaper and practical alternative to isokinetic testing in a laboratory setting. The investigators collaborated with a hospital engineer to create a portable, height-adjustable stabilising device that could accommodate each participant’s sitting height. The velcro straps on the hinged arm device provided enough elasticity to conform around any body shape or size to provide additional stability. When performing isometric strength testing with HHD in clinical practice, it is critical to ensure that the patient makes no compensatory movements, as this can significantly affect measurement accuracy. Furthermore, using a stabilising device eliminates the variable of tester strength, which can also affect reliability. In contrast to Kolber et al. [43], our device provided hand-free stabilisation, enabling the therapist to closely monitor technique and apply additional stability to the distal end of the humerus if required. Although we used a healthy cohort, any level of pain or discomfort should be considered when positioning patients with shoulder pathology in various degrees of abduction.

The reliability assessment for shoulder testing protocols for a single rater and among raters is one of the study’s strengths. We blinded raters to isometric ER strength measurements by using a third independent rater to collect and analyse results. Furthermore, we assessed and evaluated the reliability of isometric strengthening in 30° and 45° degrees of abduction.

This study had some limitations. First, we used a relatively young and asymptomatic cohort with no known shoulder or neck pathology. A healthy population was chosen to avoid the inclusion of any confounding variables that may have impacted measurement outcomes. However, future research should compare the reliability measures of healthy cohorts to those of shoulder pathology. Second, we did not compare active and passive ROM because our aim was to evaluate the reliability and measurement error of active shoulder ROM protocols. Third, because we only utilised one patient position for each type of movement and measurement tool, we cannot make any inferences as to whether different patient positions impact reliability. Four, to potentially improve measurement accuracy with digital photography we could have standardised the angle and height of the tablet device by utilising a tripod stand. Lastly, for HBB measurements we used a PSIS protocol and did not compare it with the vertebral T1 level method. We adopted van den Dolder and Roberts’ [28] modified PSIS level approach because it was easier to palpate and anatomically identify than the vertebral body [62].

## 5. Conclusions

This study confirmed the reliability of standardised shoulder ROM and strength testing protocols. According to the criteria by Portney and Watkins [47], ICC values above 0.90 are considered acceptable for clinical purposes. HBB with tape measure and isometric strength testing with HHD and a stabilisation device both meet this clinical criterion. Future studies comparing different protocols, movements, and patient positions for the same type of measurement, can provide clinicians with further confidence in applying specific testing protocols for shoulder assessment.

## Figures and Tables

**Table 1:** Characteristics of patients (n = 24).

Characteristics	Mean (SD) or Frequency (%)
Age (years)	31.2 (10.9)
BMI (kg/m^2^)	23.5 (2.4)
Sex	
Male	12 (50%)
Female	12 (50%)
Hand dominance	
Right	20 (83%)
Left	4 (17%)

BMI: body mass index, SD: standard deviation.

**Table 2:** Intra-rater reliability for rater A and B (n = 24).

	Rater A			Rater B		
Type of Measurement	ICC (95% CI)	SEM	MDC	ICC (95% CI)	SEM	MDC
**Shoulder ROM**						
*Photography*						
Forward Flexion (°)	0.86 (0.73–0.92)	2.4	5.5	0.76 (0.43–0.88)	3.5	8.3
*Tape Measure*						
HBB (cm)	0.96 (0.93–0.98)	1.0	2.3	0.94 (0.88–0.97)	1.2	2.7
**Shoulder strength**						
HHD						
ER 30° Abd (kg)	0.97 (0.96–0.98)	0.7	1.7	0.96 (0.93–0.98)	0.8	1.8
ER 45° Abd (kg)	0.97 (0.94–0.98)	0.4	0.8	0.98 (0.95–0.99)	1.1	2.5

ICC, intraclass correlation coefficient; SEM, standard error of measurement; MDC; minimal detectable change; CI, confidence interval; HBB, hand-behind-back; ER, external rotation; HHD, hand-held dynamometry; Abd, abduction, ROM; range of motion.

**Table 3:** Mean and SDs for all measurements and trial sessions (n = 24).

Type of Measurement	Rater A		Rater B	
	Session 1	Session 2	Session 1	Session 2
	Mean (SD)	Mean (SD)	Mean (SD)	Mean (SD)
**Shoulder ROM**				
Forward Flexion (°)	150.0 (6.1)	148.5 (6.5)	147.4 (6.9)	143.9 (6.7)
HBB (cm)	37.1 (5.3)	37.3 (4.6)	38.7 (4.9)	39.5 (4.7)
**Shoulder strength**				
ER 30° Abd (kg)	9.2 (3.5)	9.7 (4.0)	9.6 (3.9)	9.8 (3.9)
ER 45° Abd (kg)	9.9 (4.1)	10.4 (4.3)	15.7 (9.0)	10.3 (4.5)

SD, standard deviation; Abd, Abduction; HBB, hand-behind-back; ER, external rotation; ROM, range of motion.

**Table 4:** Inter-rater reliability (n = 24).

Type of Measurement	ICC (95% CI)	SEM	MDC
**Shoulder ROM**			
*Photography*			
Forward Flexion (°)	0.75 (0.40–0.88)	1.3	2.0
*Tape measure*			
HBB (cm)	0.91 (0.61–0.96)	0.7	1.0
**Shoulder strength**			
HHD			
ER 30° Abd (kg)	0.97 (0.95–0.98)	0.3	0.5
ER 45° Abd (kg)	0.98 (0.97–0.99)	0.6	1.4

ICC, intraclass correlation coefficient; SEM, standard error of measurement; MDC, minimal detectable change; CI, confidence interval; HBB, hand-behind-back; ER, external rotation; HHD, hand-held dynamometry; Abd, abduction, ROM, range of motion.

**Table 5:** Rater A and B shoulder measurement comparisons by arm dominance (n = 24).

Rater A Measurements	Dominant Arm	Non Dominant Arm		
	Mean (SD)	Mean (SD)	Mean Diff	*p*-Value
Forward Flexion (°)	150.1 (5.4)	148.4 (7.1)	1.6	0.057
HBB (cm)	36.0 (4.5)	38.2 (5.5)	−2.2	<0.001 *
ER 30° Abd (kg)	9.6 (3.7)	9.3 (3.8)	0.3	0.078
ER 45° Abd (kg)	10.5 (4.3)	9.8 (4.1)	0.7	<0.001 *
**Rater B measurements**	**Dominant Arm**	**Non Dominant Arm**		
	**Mean (SD)**	**Mean (SD)**	**Mean Diff**	***p*** **-Value**
Forward Flexion (°)	146.1 (6.7)	145.2 (7.1)	0.9	0.143
HBB (cm)	37.8 (4.3)	40.4 (4.9)	2.7	<0.001 *
ER 30° Abd (kg)	10.0 (3.9)	9.4 (3.9)	0.7	<0.001 *
ER 45° Abd (kg)	10.3 (4.4)	9.8 (4.2)	0.5	<0.011 *

HBB; hand-behind back; ER, external rotation; Abd, abduction; * indicates statistical significance (*p* < 0.05).

**Table 6:** Peak measurement comparisons between dominant and non-dominant arm (n = 24).

Type of Measurement	Rater A		Rater B	
	Session 1	Session 2	Session 1	Session 2
	n (%)	n (%)	n (%)	n (%)
**Peak Forward Flexion** (°)				
*Dominant arm highest*	12 (50)	14 (58)	14 (58)	13 (54)
*Non-dominant arm highest*	11 (46)	8 (33)	8 (33)	11 (46)
*Equal*	1 (4)	2 (8)	2 (8)	0 (0)
**Peak Hand-Behind-Back (cm)**				
*Dominant arm highest*	18 (75)	18 (75)	22 (75)	18 (75)
*Non-dominant arm highest*	6 (25)	6 (25)	6 (25)	5 (21)
*Equal*	0 (0)	0 (0)	1 (4)	1 (4)
**Peak ER strength** **at 30**° **Abd (kg)**				
*Dominant arm highest*	15 (63)	14 (58)	18 (75)	19 (80)
*Non-dominant arm highest*	8 (33)	10 (42)	6 (25)	4 (17)
*Equal*	1 (4)	0 (0)	0 (0)	1 (4)
**Peak ER strength at 45**° **Abd (kg)**				
*Dominant arm highest*	17 (71)	16 (67)	17 (71)	14 (58)
*Non-dominant arm highest*	7 (29)	7 (29)	7 (29)	10 (42)
*Equal*	0 (0)	1 (4)	0 (0)	0 (0)
**Peak ER strength (at 30**° **and 45**° **Abd) (kg)**				
*Dominant arm highest*	13 (54)	11 (46)	12 (50)	12 (50)
*Non-dominant arm highest*	4 (17)	5 (21)	1 (4)	3 (13)
*Stronger in different arms*	7 (29)	8 (33)	11 (46)	9 (38)

ER, external rotation; Abd, abduction.

## Data Availability

Data available in a publicly accessible repository: https://zenodo.org/record/6960822#.YutD_HZByUk (accessed on 8 August 2022).

## References

[1] Streiner D.L., Norman G.R. (2008). Health Measurement Scales: A Practical Guide to Their Development and Use.

[2] Stark T., Walker B., Phillips J.K., Fejer R., Beck R. (2011). Hand-held dynamometry correlation with the gold standard isokinetic dynamometry: A systematic review. PM&R.

[3] Gajdosik R.L., Bohannon R.W. (1987). Clinical measurement of range of motion. Review of goniometry emphasizing reliability and validity. Phys. Ther..

[4] Reese N., Bandy W. (2002). Joint Range of Movement and Muscle Testing.

[5] Boone D.C., Azen S.P., Lin C.M., Spence C., Baron C., Lee L. (1978). Reliability of goniometric measurements. Phys. Ther..

[6] Terwee C.B., de Winter A.F., Scholten R.J., Jans M.P., Devillé W., van Schaardenburg D., Bouter L.M. (2005). Interobserver reproducibility of the visual estimation of range of motion of the shoulder. Arch. Phys. Med. Rehabil..

[7] Croft P., Pope D., Boswell R., Rigby A., Silman A. (1994). Observer variability in measuring elevation and external rotation of the shoulder. Primary Care Rheumatology Society Shoulder Study Group. Br. J. Rheumatol..

[8] Kolber M.J., Vega F., Widmayer K., Cheng M.S. (2011). The reliability and minimal detectable change of shoulder mobility measurements using a digital inclinometer. Physiother. Theory Pract..

[9] Green S., Buchbinder R., Forbes A., Bellamy N. (1998). A standardized protocol for measurement of range of movement of the shoulder using the Plurimeter-V inclinometer and assessment of its intrarater and interrater reliability. Arthritis Care Res..

[10] Nadeau S., Kovacs S., Gravel D., Piotte F., Moffet H., Gagnon D., Hébert L.J. (2007). Active movement measurements of the shoulder girdle in healthy subjects with goniometer and tape measure techniques: A study on reliability and validity. Physiother. Theory Pract..

[11] Han S.H., Oh K.S., Han K.J., Jo J., Lee D.H. (2012). Accuracy of measuring tape and vertebral-level methods to determine shoulder internal rotation. Clin. Orthop. Relat. Res..

[12] Ginn K.A., Herbert R.D., Khouw W., Lee R. (1997). A randomized, controlled clinical trial of a treatment for shoulder pain. Phys. Ther..

[13] Chen J.F., Ginn K.A., Herbert R.D. (2009). Passive mobilisation of shoulder region joints plus advice and exercise does not reduce pain and disability more than advice and exercise alone: A randomised trial. Aust. J. Physiother..

[14] Rajkumar A., Vulpi F., Bethi S.R., Wazir H.K., Raghavan P., Kapila V. (2020). Wearable Inertial Sensors for Range of Motion Assessment. IEEE Sens. J..

[15] Rigoni M., Gill S., Babazadeh S., Elsewaisy O., Gillies H., Nguyen N., Pathirana P.N., Page R. (2019). Assessment of Shoulder Range of Motion Using a Wireless Inertial Motion Capture Device-A Validation Study. Sensors.

[16] Werner B.C., Holzgrefe R.E., Griffin J.W., Lyons M.L., Cosgrove C.T., Hart J.M., Brockmeier S.F. (2014). Validation of an innovative method of shoulder range-of-motion measurement using a smartphone clinometer application. J. Shoulder Elbow Surg..

[17] Shin S.H., Ro du H., Lee O.S., Oh J.H., Kim S.H. (2012). Within-day reliability of shoulder range of motion measurement with a smartphone. Man Ther..

[18] Wilson J.D., Khan-Perez J., Marley D., Buttress S., Walton M., Li B., Roy B. (2017). Can shoulder range of movement be measured accurately using the Microsoft Kinect sensor plus Medical Interactive Recovery Assistant (MIRA) software?. J. Shoulder Elbow Surg..

[19] Overbeek C.L., Geurkink T.H., de Groot F.A., Klop I., Nagels J., Nelissen R.G., de Groot J.H. (2021). Shoulder movement complexity in the aging shoulder: A cross-sectional analysis and reliability assessment. J. Orthop. Res..

[20] Wilk K.E., Reinold M.M., Macrina L.C., Porterfield R., Devine K.M., Suarez K., Andrews J.R. (2009). Glenohumeral internal rotation measurements differ depending on stabilization techniques. Sports Health.

[21] Hayes K., Walton J.R., Szomor Z.R., Murrell G.A. (2001). Reliability of five methods for assessing shoulder range of motion. Aust. J. Physiother..

[22] Crasto J.A., Sayari A.J., Gray R.R., Askari M. (2015). Comparative analysis of photograph-based clinical goniometry to standard techniques. Hand.

[23] Blonna D., Zarkadas P.C., Fitzsimmons J.S., O’Driscoll S.W. (2012). Validation of a photography-based goniometry method for measuring joint range of motion. J. Shoulder Elbow Surg..

[24] Cuesta-Vargas A.I., Roldán-Jiménez C. (2016). Validity and reliability of arm abduction angle measured on smartphone: A cross-sectional study. BMC Musculoskelet. Disord..

[25] Russo R.R., Burn M.B., Ismaily S.K., Gerrie B.J., Han S., Alexander J., Lenherr C., Noble P.C., Harris J.D., McCulloch P.C. (2018). Is digital photography an accurate and precise method for measuring range of motion of the shoulder and elbow?. J. Orthop. Sci..

[26] Edwards T.B., Bostick R.D., Greene C.C., Baratta R.V., Drez D. (2002). Interobserver and intraobserver reliability of the measurement of shoulder internal rotation by vertebral level. J. Shoulder Elbow Surg..

[27] Hoving J.L., Buchbinder R., Green S., Forbes A., Bellamy N., Brand C., Buchanan R., Hall S., Patrick M., Ryan P. (2002). How reliably do rheumatologists measure shoulder movement?. Ann. Rheum. Dis..

[28] Van den Dolder P.A., Roberts D.L. (2003). A trial into the effectiveness of soft tissue massage in the treatment of shoulder pain. Aust. J. Physiother..

[29] van den Dolder P.A., Ferreira P.H., Refshauge K. (2014). Intra- and inter-rater reliability of a modified measure of hand behind back range of motion. Man Ther..

[30] Hislop H.J., Montgomery J. (2007). Daniels & Worthingham's Muscle Testing: Techniques of Manual Examination.

[31] Nagatomi T., Mae T., Nagafuchi T., Yamada S.I., Nagai K., Yoneda M. (2017). Shoulder manual muscle resistance test cannot fully detect muscle weakness. Knee Surg. Sports Traumatol. Arthrosc..

[32] Hayes K., Walton J.R., Szomor Z.L., Murrell G.A. (2002). Reliability of 3 methods for assessing shoulder strength. J. Shoulder Elbow Surg..

[33] Karabay D., Yesilyaprak S.S., Sahiner Picak G. (2020). Reliability and validity of eccentric strength measurement of the shoulder abductor muscles using a hand-held dynamometer. Phys. Ther. Sport.

[34] Johansson F.R., Skillgate E., Lapauw M.L., Clijmans D., Deneulin V.P., Palmans T., Engineer H.K., Cools A.M. (2015). Measuring Eccentric Strength of the Shoulder External Rotators Using a Handheld Dynamometer: Reliability and Validity. J. Athl. Train..

[35] Sullivan S.J., Chesley A., Hebert G., McFaull S., Scullion D. (1988). The validity and reliability of hand-held dynamometry in assessing isometric external rotator performance. J. Orthop. Sports Phys. Ther..

[36] Holt K.L., Raper D.P., Boettcher C.E., Waddington G.S., Drew M.K. (2016). Hand-held dynamometry strength measures for internal and external rotation demonstrate superior reliability, lower minimal detectable change and higher correlation to isokinetic dynamometry than externally-fixed dynamometry of the shoulder. Phys. Ther. Sport.

[37] Wikholm J.B., Bohannon R.W. (1991). Hand-held dynamometer measurements: Tester strength makes a difference. J. Orthop. Sport. Phys. Ther..

[38] Stone C.A., Nolan B., Lawlor P.G., Kenny R.A. (2011). Hand-held dynamometry: Tester strength is paramount, even in frail populations. J. Rehabil. Med..

[39] Wadsworth C.T., Krishnan R., Sear M., Harrold J., Nielsen D.H. (1987). Intrarater reliability of manual muscle testing and hand-held dynametric muscle testing. Phys. Ther..

[40] Reed R.L., Den Hartog R., Yochum K., Pearlmutter L., Ruttinger A.C., Mooradian A.D. (1993). A comparison of hand-held isometric strength measurement with isokinetic muscle strength measurement in the elderly. J. Am. Geriatr. Soc..

[41] Agre J.C., Magness J.L., Hull S.Z., Wright K.C., Baxter T.L., Patterson R., Stradel L. (1987). Strength testing with a portable dynamometer: Reliability for upper and lower extremities. Arch. Phys. Med. Rehabil..

[42] Wang C.Y., Olson S.L., Protas E.J. (2002). Test-retest strength reliability: Hand-held dynamometry in community-dwelling elderly fallers. Arch. Phys. Med. Rehabil..

[43] Kolber M.J., Beekhuizen K., Cheng M.S., Fiebert I.M. (2007). The reliability of hand-held dynamometry in measuring isometric strength of the shoulder internal and external rotator musculature using a stabilization device. Physiother. Theory Pract..

[44] Makhni E.C., Steinhaus M.E., Morrow Z.S., Jobin C.M., Verma N.N., Cole B.J., Bach B.R. (2015). Outcomes assessment in rotator cuff pathology: What are we measuring?. J. Shoulder Elbow Surg..

[45] Walter S.D., Eliasziw M., Donner A. (1998). Sample size and optimal designs for reliability studies. Stat. Med..

[46] De Vet H.C., Terwee C.B., Knol D.L., Bouter L.M. (2006). When to use agreement versus reliability measures. J. Clin. Epidemiol..

[47] Portney L.G., Watkins M.P. (2015). Foundations of Clinical Research: Applications to Practice.

[48] Weir J.P. (2005). Quantifying Test-Retest Reliability Using the Intraclass Correlation Coefficient and the SEM. J. Strength Cond. Res..

[49] Bland J.M., Altman D. (1986). Statistical methods for assessing agreement between two methods of clinical measurement. Lancet.

[50] De Winter A.F., Heemskerk M.A., Terwee C.B., Jans M.P., Devillé W., van Schaardenburg D.J., Scholten R.J., Bouter L.M. (2004). Inter-observer reproducibility of measurements of range of motion in patients with shoulder pain using a digital inclinometer. BMC Musculoskelet. Disord..

[51] Greenfield B.H., Donatelli R., Wooden M.J., Wilkes J. (1990). Isokinetic evaluation of shoulder rotational strength between the plane of scapula and the frontal plane. Am. J. Sports Med..

[52] Rathbun J., Macnab I. (1970). The microvascular pattern of the rotator cuff. J. Bone Jt. Surg..

[53] Poppen N., Walker P.S. (1978). Forces at the glenohumeral joint in abduction. Clin. Orthop..

[54] Edouard P., Samozino P., Julia M., Gleizes Cervera S., Vanbiervliet W., Calmels P., Gremeaux V. (2011). Reliability of isokinetic assessment of shoulder-rotator strength: A systematic review of the effect of position. J. Sport Rehabil..

[55] Schrama P.P., Stenneberg M.S., Lucas C., van Trijffel E. (2014). Intraexaminer reliability of hand-held dynamometry in the upper extremity: A systematic review. Arch. Phys. Med. Rehabil..

[56] Mullaney M.J., McHugh M.P., Johnson C.P., Tyler T.F. (2010). Reliability of shoulder range of motion comparing a goniometer to a digital level. Physiother. Theory Pract..

[57] Correll S., Field J., Hutchinson H., Mickevicius G., Fitzsimmons A., Smoot B. (2018). Reliability and validity of the halo digital goniometer for shoulder range of motion in healthy subjects. Int. J. Sports Phys. Ther..

[58] Muir S.W., Corea C.L., Beaupre L. (2010). Evaluating change in clinical status: Reliability and measures of agreement for the assessment of glenohumeral range of motion. N. Am. J. Sports Phys. Ther..

[59] Shimizu H., Saito T., Kouno C., Shimoura K., Kawabe R., Shinohara Y., Mukaiyama K., Changyu C., Kato M., Nagai-Tanima M. (2022). Validity and reliability of a smartphone application for self-measurement of active shoulder range of motion in a standing position among healthy adults. JSES Int..

[60] Lin Y.C., Tsai Y.J., Hsu Y.L., Yen M.H., Wang J.S. (2021). Assessment of Shoulder Range of Motion Using a Wearable Inertial Sensor Network. IEEE Sens. J..

[61] Özsoy U., Yıldırım Y., Karaşin S., Şekerci R., Süzen L.B. (2022). Reliability and agreement of Azure Kinect and Kinect v2 depth sensors in the shoulder joint ROM estimation. J Shoulder Elbow Surg..

[62] Fryer G., McPherson H.C., O’Keefe P. (2005). The effect of training on the inter-examiner and intra-examiner reliability of the seated flexion test and assessment of pelvic anatomical landmarks with palpation. Int. J. Osteopath. Med..

